# A comprehensive analysis comparing the eighth AJCC gastric cancer pathological classification to the seventh, sixth, and fifth editions

**DOI:** 10.1002/cam4.1230

**Published:** 2017-11-03

**Authors:** Sharvesh Raj Seeruttun, Shuqiang Yuan, Haibo Qiu, Yun Huang, Yuanfang Li, Yao Liang, Yuanxiang Guan, Youqing Zhan, Wei Li, Yingbo Chen, Xiaowei Sun, Dazhi Xu, Zhiwei Zhou

**Affiliations:** ^1^ Department of Gastric Surgery Sun Yat‐sen University Cancer Center Guangzhou China; ^2^ Department of Medical Statistics and Epidemiology Sun Yat‐sen University Guangzhou China

**Keywords:** AJCC, classification, gastric cancer, staging, TNM

## Abstract

To perform a comprehensive analysis comparing the prognostic and discriminative ability of the eighth AJCC gastric cancer (GC) pathological classification to that of the seventh, sixth and fifth editions, and secondly to assess their long‐term significance. Patients who had undergone R0 gastrectomy were identified and restaged accordingly. To evaluate and confirm any difference in prognostic ability between the competing editions, the Akaike information criterion (AIC) and Bayesian information criterion (BIC) were computed and compared since both have different analytic strengths. The area under the curve (AUC) with 95% CI based on the time‐dependent receiver‐operating characteristics analyses were also calculated to assess any change in prognostic rankings from the first to tenth postoperative year. The rankings calculated by both statistical methods showed similar results, in which the seventh edition was identified as possessing the best prognostic ability. Additionally, these ranks were found to remain consistent over the ten postoperative years, but demonstrated no clinical significance as their respective 95% CIs calculated by the AIC, BIC, and AUC were found to overlap. However, the more detailed staging classifications of the eighth edition was shown to display the best prognostic demarcation for stratifying patients with higher‐staged disease. This study thereby identified the eighth AJCC GC edition to possess similar long‐term prognostic ability as to its previous three editions but contrastingly demonstrated the best distinctive ability for stratifying overall survival and can thus be considered as being clinically more reliable.

## Introduction

Gastric cancer (GC) is the second leading cause of cancer‐related death worldwide, with China having the largest pool of advanced GC cases 1, for which high‐level surgical and medical prowess are required to improve survival. Patients often travel long distances to specialized cancer centers mainly for surgeries but often prefer their local hospitals for adjuvant therapies. As such, to enable a standardized treatment, staging of the disease is therefore the fundamental common language between surgical and medical oncologists.

The most recognized evidence‐based GC staging system in practice is the tumor‐node‐metastasis (TNM) concept from the American Joint Committee on Cancer (AJCC). Since the release of its first edition in 1977 2, it has been updated every few years based on new breakthroughs in oncology.

Starting from the fifth AJCC GC edition, the anatomic nodal classification was discontinued and reporting the number of surgically retrieved lymph nodes (LNs) became the proposed standard 3. The sixth edition had only minor updates that did not influence the main staging of the disease 4. As such, in this study they were considered alike and labeled as the fifth/sixth edition. However, the seventh edition brought considerable modifications to the pathological classification of the depth of tumor invasion (pT) and completely redefined the classification of metastasized lymph nodes (pN) 5.

The eighth edition was recently released and includes the implementation of a clinical stage group, a postneoadjuvant stage group and several substantial revisions to the pathological stage group (pTNM) 6. The major changes hallmarked in this new edition are firstly, separating the pN3ab regional lymph nodes from the seventh edition into pN3a and pN3b in its main stage groupings. Secondly, the anatomic boundary demarcating esophageal and gastric cancer for tumors arising at the esophagogastric junction (EGJ) was adjusted from 5 cm to 2 cm.

Previous publications comparing the prognostic and discriminative abilities between the seventh and the sixth gastric cancer editions lacked detailed‐enough analyses, which might have contributed to the conflicting results published 7, 8, 9, 10, 11. In addition, since the implemented updates mainly affect patients with higher‐staged disease and Chinese patients are comparatively diagnosed at a more advanced stage, our primary aim was to use our Chinese cohort to perform a comprehensive analysis comparing the discriminative and prognostic ability of the eighth AJCC GC pathological classification to that of the seventh and fifth/sixth editions and secondly, to assess their long‐term significance.

## Methods

### Patient cohort

From the prospectively recorded database of the Gastric Surgical Division of Sun Yat‐sen University Cancer Center, Guangzhou, China, a total of 2151 GC patients who had undergone surgical resection from January 1990 to December 2013 were identified. The patients’ data were screened according to the following inclusion criteria: (1) thorough preoperative examinations including histologically confirmed adenocarcinoma of the stomach with no previous cancer history; (2) radical gastrectomy with radiological/pathological examination confirming the absence of synchronous or metachronous malignancies; (3) no neoadjuvant therapies; and (4) complete clinicopathological data to enable restaging according to the different AJCC TNM classifications.

The patients were followed every 3 months in the first 2 years after surgery, every 6 months for the next 3 years and then annually afterwards. Clinical examinations including general complete physical, hematological, and radiological tests were performed as required. The last day of follow‐up was February 2017.

### Surgical treatment and pathological classification

Patients with endoscopic or radiologic confirmation of gastric cancer involving the esophagus are often treated at the Thoracic Department of our Cancer Center. For consistency in surgical treatment, they were not included in this study. Expert gastric surgeons, each with an individual experience of at least 2000 gastrectomies, performed the surgical procedures according to the Japanese Gastric Cancer Association guidelines.

In this study, early, middle, and locally advanced disease referred to stages IA, IB to IIIA and IIIB to IIIC according to the eighth AJCC GC staging system, respectively and, combined resection referred to dissection of the spleen, pancreas and/or liver in addition to gastrectomies for achieving R0 resection.

All specimens were processed postoperatively by one of the operating surgeons before being submitted to expert pathologists whereby they were staged according to the most recent AJCC TNM classification at that time. For this study, each case was restaged according to the fifth/sixth, seventh and eighth AJCC GC pathological staging system. This retrospective study received the approval of the Institutional Review Board of the Ethical Committee of Sun Yat‐sen University Cancer Center, and upon final analysis, 1797 patients were observed to match the inclusion criteria.

### Statistical analysis

Survival time was calculated from the date of surgery till the last day of follow‐up or tumor‐related death. Kaplan–Meier was used to calculate survival time and for statistical comparison of prognosis. The Cox proportional hazard model with forward stepwise regression was used to compute three separate multivariate analyses, namely, Multivariate 1, 2 and 3, which consisted of the parameters found to be significant in the univariate analysis for the fifth/sixth, seventh and eighth editions, respectively.

To identify the model with the best predictive ability, their corresponding Akaike information criterion (AIC) and Bayesian information criterion (BIC) with 95% CIs from bootstrapping of the original data 12 were also computed.

Next, to assess whether their prognostic abilities would change over the years, we performed time‐dependent receiver operating characteristic (ROC) analyses of the area‐under‐curve (AUC), based on the predictive value of multivariate analyses, with 95% CI for the first, second, third, fourth, fifth, and tenth postoperative years. Their discriminative abilities were assessed by analyzing and comparing the range and gap of their survival curves.

Statistical analyses were performed using SPSS software (version 22.0, SPSS Inc., Chicago, IL) and R statistical software (version 3.3.1, the *R* Foundation for Statistical Computing). A *P*‐value less than 0.05 (2‐sided) was considered to be statistically significant.

## Results

### Patient characteristics

Patients with stage III disease, according to the eighth edition, amounted to 51% (*n* = 916) of the cases. The mean age of the study population was 56.8 years (range, 16–90 years), and the 5‐year overall survival rate was 65.8 ± 2.876% (rate ± SD). In this study, there was a total of 37,682 LNs examined for which an average number of 21 LNs were recorded per patient. With regard to the 131 patients who had combined resection, a small percentage of them (*n* = 13; 9.9%) experienced postoperative complications, among which 109 (83.2%) had tumors greater than 4.5 cm (ranging from 5 cm to 18 cm), 89.3% being locally advanced and with the majority of them (35.1%) located in the upper third of the stomach. The median follow‐up time was 45 months (range, 1–259 months).

### Change in patient distribution

No change in distribution was observed in stage IA of the three editions. Patients in stage IB (*n* = 203) of the fifth/sixth edition were reclassified to stage IB (*n* = 107) and IIA (*n* = 96) in the eighth edition, those in stage II (*n* = 461) were reclassified to stage IIA (*n* = 34), IIB (*n* = 368) and IIIA (*n* = 59), and those in stage IIIA (*n* = 502) were reclassified to stage IIIA (*n* = 441) and IIIB (*n* = 61) in the new edition. Finally, patients in stage IIIB (*n* = 157) of the fifth/sixth edition were reclassified to stage IIIB (*n* = 154) and IIIC (*n* = 3), and those in stage IV (*n* = 198) were reclassified to stage IIIB (*n* = 34) and IIIC (*n* = 164), respectively. A large proportion of the patients (*n* = 1346; 74.9%) were upstaged in the new GC edition, and no down‐staging between these two classifications was observed.

From the seventh to the eighth edition, no change in distribution was found for 76% of the patients. A small percentage (*n* = 5; 1.4%) of patients from stage IIB in the seventh edition was reclassified to stage IIIB in the new edition. Those in stage IIIB (*n* = 332) were reclassified to stage IIIA (*n* = 232), IIIB (*n* = 75) and IIIC (*n* = 25), while those in stage IIIC (*n* = 311) were reclassified to stage IIIB (*n* = 169) and IIIC (*n* = 142), respectively. In all, 1.7% and 22.3% of patients from the seventh edition were up‐staged and down‐staged in the eighth edition, respectively.

### Survival analysis

**Figure 1:**
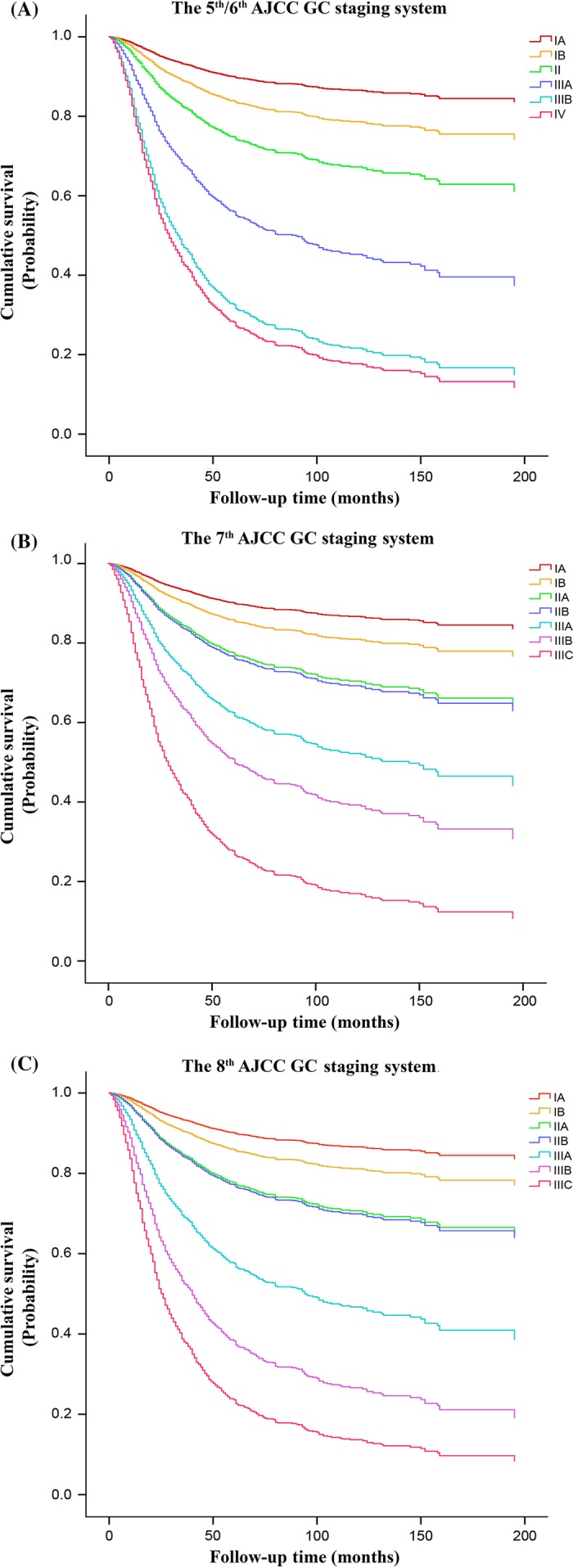
Distribution of the survival curves according to the American Joint Committee on Cancer (AJCC) TNM classification of the (A) fifth/sixth, (B) seventh, and (C) eighth edition.

Of the eighteen clinicopathological factors analyzed in univariate analysis, only sex showed no correlation with survival (Table 1). A continuous decrease in 5‐year overall survival (OS) rate with an increase in increment of the pTNM classification as well as a gradual increase in their HR values and larger range of 95% confidence intervals (Table 1 and Fig. 1A–C; *P *<* *0.001) demonstrated the progressive improvement in demarcation of prognoses between the stages from the fifth/sixth to the eighth edition.

**Table 1:** Correlation of patients demographics and clinical characteristics with survival

Characteristics	No. of cases	Cases (%)	5‐year OS (%)	HR	95% CI	*P*‐value
Sex						0.554
Male	1207	67.2	62.6	Ref		
Female	590	32.8	60.9	1.049	0.895–1.230	
Age (years)						<0.001
≤64	1318	73.3	65.1	Ref		
>64	479	26.7	53.9	1.574	1.346‐1.841	
Tumor location						<0.001
>1/3 of stomach	161	9.0	39.6	Ref		
Upper 1/3	570	32.0	51.1	0.730	0.576–0.923	
Middle 1/3	155	8.6	71.8	0.376	0.265–0.534	
Lower 1/3	911	50.7	71.1	0.352	0.277–0.447	
Tumor size (cm)						<0.001
<4.5	806	44.9	74.8	Ref		
4.5 ≤ *T *<* *8.0	617	34.3	58.1	1.816	1.515–2.1977	
≥8.0	374	20.8	41.3	2.980	2.468–3.598	
Bormann type						<0.001
I	143	8.0	84.2	Ref		
II	586	32.6	71.1	2.108	1.356–3.277	
III	986	54.9	55.4	3.480	2.267–5.342	
IV	82	4.5	35.5	6.096	3.684–10.089	
Differentiation						0.039
High/moderate	360	20.0	65.8	Ref		
Poor/undifferentiated/signet cell	1437	80.0	61.1	1.227	1.010–1.491	
Type of gastrectomy						<0.001
Proximal	496	27.6	50.3	Ref		
Distal	987	54.9	71.3	0.467	0.395–0.552	
Total	314	17.5	50.0	0.971	0.792–1.190	
Combined resection						<0.001
No	1666	92.7	64.2	Ref		
Yes	131	7.3	35.9	2.338	1.865–2.931	
Postoperative complication						0.029
No	1724	95.9	62.7	Ref		
Yes	73	4.1	46.6	1.464	1.037–2.067	
Retrieved lymph nodes						<0.001
<16	749	41.7	55.7	Ref		
≥16	1048	58.3	66.7	0.649	0.558–0.754	
Adjuvant chemotherapy						<0.001
No	1321	73.5	64.3	Ref		
Yes	476	26.5	55.8	1.335	1.139–1.564	
Fifth/Sixth edition pT						<0.001
T1	399	22.2	86.9	Ref		
T2	383	21.3	71.0	2.794	1.996–3.910	
T3	904	50.3	51.0	5.184	3.850–6.981	
T4	111	6.2	35.6	8.494	5.910–12.206	
Fifth/Sixth edition pN						<0.001
N0	657	36.6	81.8	Ref		
N1	723	40.2	56.3	2.797	2.288–3.419	
N2	282	15.7	44.1	3.965	3.140–5.007	
N3	135	7.5	30.9	5.227	3.972–6.877	
Seventh/Eighth edition pT						<0.001
T1a	271	15.1	83.9	Ref		
T1b	128	7.1	93.0	0.460	0.223–0.949	
T2	106	5.9	73.5	2.305	1.485–3.577	
T3	277	15.4	69.0	2.281	1.550–3.358	
T4a	903	50.3	50.9	4.256	3.068–5.902	
T4b	112	6.2	36.3	6.844	4.647–10.081	
Seventh/Eighth edition pN						<0.001
N0	657	36.6	81.8	Ref		
N1	381	21.2	63.5	2.289	1.811–2.894	
N2	342	19.0	48.7	3.412	2.729–4.267	
N3a	282	15.7	41.4	3.976	3.147–5.018	
N3b	135	7.5	30.9	5.239	3.981–6.893	
Fifth/Sixth edition (pTNM)						<0.001
IA	276	15.4	91.2	Ref		
IB	203	11.3	83.2	2.018	1.215–3.352	
II	461	25.7	70.2	3.857	2.524–5.893	
IIIA	502	27.9	49.5	7.812	5.182–11.775	
IIIB	157	8.7	36.4	10.608	6.828–16.482	
IV	198	11.0	33.1	11.920	7.760–18.310	
Seventh edition (pTNM)						<0.001
IA	276	15.4	91.2	Ref		
IB	107	6.0	85.9	1.975	1.105–3.531	
IIA	130	7.2	76.5	2.906	1.716–4.922	
IIB	373	20.8	71.5	3.629	2.354–5.593	
IIIA	268	14.9	55.2	6.567	4.271–10.096	
IIIB	332	18.5	47.5	8.282	5.448–12.589	
IIIC	311	17.3	32.4	12.079	7.967–18.314	
Eighth edition (pTNM)						<0.001
IA	276	15.4	91.2	Ref		
IB	107	6.0	85.9	1.977	1.106–3.534	
IIA	130	7.2	76.5	2.906	1.716–4.923	
IIB	368	20.5	71.7	3.607	2.339–5.563	
IIIA	500	27.8	49.4	7.761	5.149–11.700	
IIIB	249	13.9	44.2	8.992	5.868–13.779	
IIIC	167	9.3	31.3	12.637	8.172–19.543	

GC, gastric cancer; HR, hazard ratio; CI, confidence interval; OS, overall survival; T, tumor; Ref, reference; pT, pathological depth of tumor invasion; pN, pathological nodal metastasis; pTNM, pathological tumor‐node‐metastasis classification of the respective gastric cancer editions.

**Figure 2:**
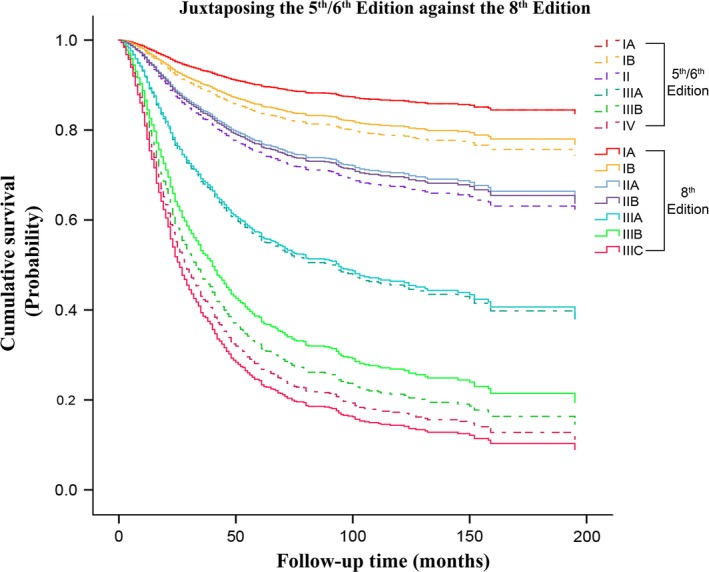
A juxtaposed comparison of the fifth/sixth edition against the eighth edition showing the differences in overall survival. The gap between the survival curves of the eighth edition are better distributed, showing higher discriminatory ability, particularly from stage IIB to IIIC.

**Figure 3:**
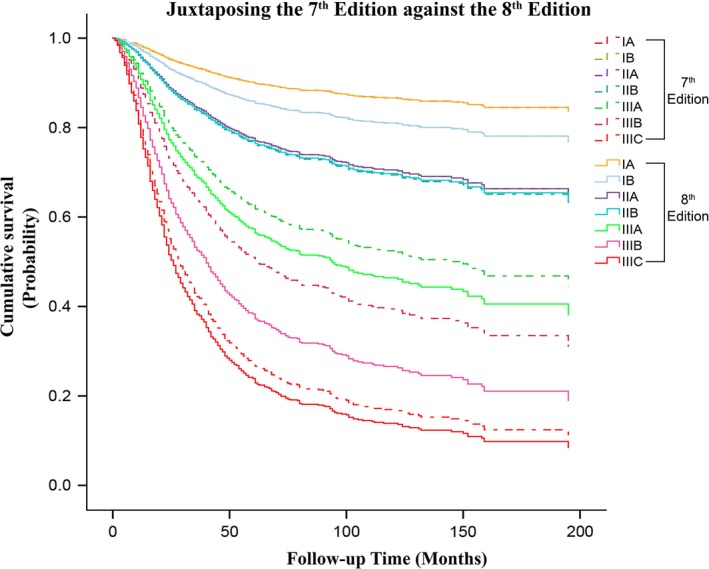
A juxtaposed comparison of the seventh against the eighth edition showing the differences in overall survival. The survival curves in the eighth edition demonstrate a greater discriminatory ability for differentiating between patients with higher stage disease, particularly for stage IIIA to IIIC.

**Figure 4:**
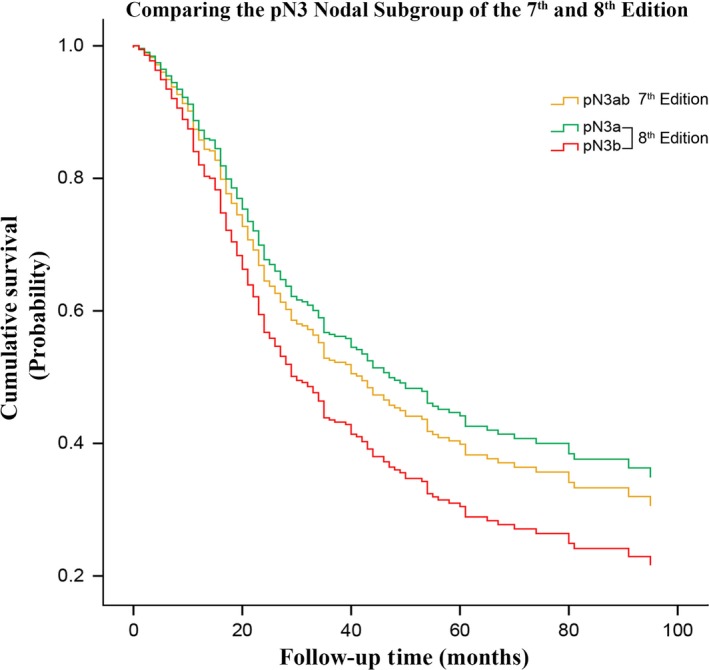
A side‐by‐side comparison illustrating the survival difference existing between patients in nodal group pN3a and pN3b compared to when classified as pN3ab.

Also, the range of 5‐year survival for the eighth edition (91.2–31.3%) were found to be progressively wider from that of the fifth/sixth (91.2–33.1%) and seventh edition (91.2–32.4%), indicating that it possesses a larger area for stratification of gastric cancer patients. As illustrated in Figures 2 and 3, this improvement was especially noted between the middle and locally advanced stages of the eighth edition against the fifth/sixth and seventh edition, respectively. Additionally, the apparent differences in survival observed from stage IIB to IIIC between the seventh and eighth edition can be primarily attributable to the different survival rates expressed by patients having pN3a and pN3b nodal disease as compared to when they were merged together as pN3ab in the seventh edition (Fig. 4).

However, although the distance between the survival curves of stage IIA and IIB in the eighth and seventh editions were relatively small, no intersection was observed, and they also expressed comparatively different 5‐year OS rates, 76.5% versus 71.7% and 76.5% versus 71.5%, respectively. A highly detailed illustration of the different combinations of pT/pN (Table 2) showed great monotonicity (continuous decrease in survival with increasing stage) and distinctiveness (difference in survival between the monotonic stages) from the old to the new edition, even though there was increased complexity of staging with each updated version. Of note, this trend could not be observed for patients with pT2 disease in the seventh and eighth editions due to their relatively low number of cases.

**Table 2:**
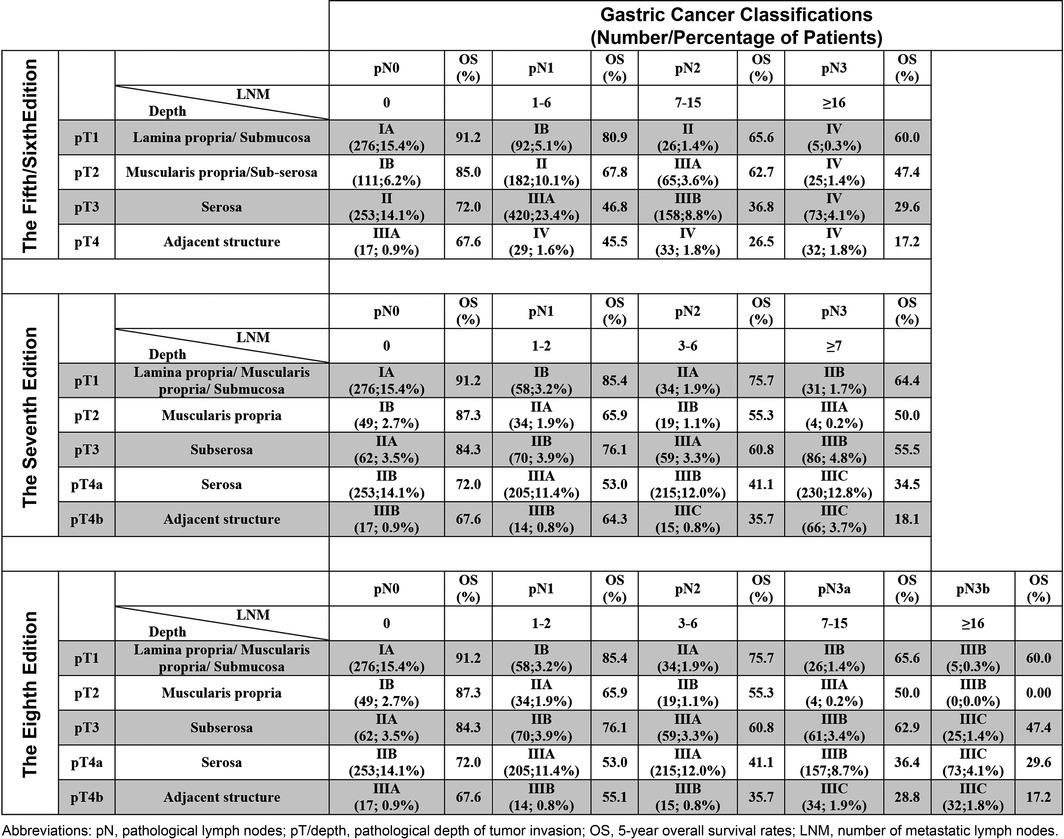
The number and percentage of patients with their corresponding 5‐year overall survival rates for the pT/pN combinations of the fifth/sixth, seventh and eighth AJCC TNM gastric cancer editions respectively

Furthermore, three different multivariate analyses were performed for each of the different AJCC GC editions and the clinical parameters found to be independently associated with survival (favorable characteristics in parentheses; Table 3) were age (≤64; *P *<* *0.001), tumor location (lower third; *P *<* *0.001), tumor size (<4.5 cm; *P *=* *0.002), and total number of retrieved LN (≥16; *P *<* *0.001).

**Table 3:** Multivariate analyses of factors associated with 5‐year overall survival for the fifth/sixth, seventh, and eighth edition

Characteristics	Multivariate analysis 1^a^			Multivariate analysis 2^b^			Multivariate analysis 3^c^		
	HR	95% CI	*P*‐value	HR	95% CI	*P*‐value	HR	95% CI	*P‐* value
Age (years)									
≤64	Ref			Ref			Ref		
>64	1.439	1.227–1.688	<0.001	1.447	1.234–1.697	<0.001	1.421	1.212–1.665	<0.001
Tumor location						<0.001			<0.001
>1/3 of stomach	Ref		<0.001	Ref		<0.001	Ref		<0.001
Upper 1/3	0.948	0.730–1.232	0.691	0.930	0.717–1.207	0.585	0.988	0.759–1.287	0.930
Middle 1/3	0.636	0.440–0.919	0.016	0.643	0.444–0.931	0.019	0.632	0.437–0.913	0.014
Lower 1/3	0.569	0.441–0.734	<0.001	0.572	0.443–0.738	<0.001	0.579	0.448–0.747	<0.001
Tumor size (cm)									
<4.5	Ref		0.002	Ref		0.002	Ref		0.003
4.5 ≤ *T *<* *8.0	1.092	0.903–1.321	0.364	1.067	0.882–1.290	0.507	1.089	0.900–1.317	0.380
≥8.0	1.409	1.143–1.738	0.001	1.411	1.145–1.738	0.001	1.422	1.154–1.753	0.001
Total LN retrieved	0.497	0.416–0.593	<0.001	0.473	0.397–0.563	<0.001	0.465	0.387–0.557	<0.001
The fifth/sixth edition (pTNM)									
IA	Ref		<0.001	Ref		<0.001	Ref		<0.001
IB	1.665	0.998–2.776	0.051	1.480	0.825–2.654	0.188	1.451	0.809–2.603	0.212
II	2.750	1.782–4.245	<0.001	2.454	1.441–4.718	<0.001	2.413	1.417–4.109	0.001
IIIA	5.495	3.596–8.396	<0.001	2.572	1.652–4.006	<0.001	2.488	1.596–3.877	<0.001
IIIB	10.610	6.691–16.823	<0.001	4.546	2.922–7.073	<0.001	5.290	3.461–8.086	<0.001
IV	11.990	7.604–18.907	<0.001	6.547	4.245–10.097	<0.001	9.203	5.889–14.380	<0.001
				12.416	7.983–19.312	<0.001	13.826	8.669–22.051	<0.001

HR, hazard ration; CI, confidence interval; ref, reference; T, tumor size; LN, lymph nodes; pTNM, pathological tumor‐node‐metastasis classification.

a Multivariate analysis 1: Clinicopathological factors showing significance in univariate analysis and the stages of the fifth/sixth edition, excluding the seventh and eighth edition stages.

b Multivariate analysis 2: Clinicopathological factors showing significance in univariate analysis and the stages of the seventh edition, excluding the fifth/sixth and eighth edition stage.

c Multivariate analysis 3: Clinicopathological factors showing significance in univariate analysis and the stages of the eighth edition, excluding the seventh and fifth/sixth edition stage.

### Prognostic performance

Table 4 illustrates the prognostic performance of the competing AJCC staging editions based on the calculations of the two different statistical methods. The best prognostic performance is determined by the lowest AIC and BIC value. As shown, the seventh edition was identified as being superior over the fifth/sixth and the eighth edition, by that were ranked as second and third, respectively. Of note, considerable overlapping of their corresponding confidence intervals (CI) was also observed.

**Table 4:** The AIC and BIC of the different AJCC gastric cancer staging editions

	Fifth/Sixth edition		Seventh edition		Eighth edition	
	Value	95% CI	Value	95% CI	Value	95% CI
AIC	9326.4	8798–9890	9315.7	8805–9891	9328.1	8792–9905
BIC	9381.0	8840–9931	9374.8	8852–9894	9387.2	8838–9947

AIC, Akaike information criterion; BIC, Bayesian information criterion; CI, confidence interval.

Furthermore, to investigate whether the above‐mentioned prognostic ranking would change over time, the AUC values from time‐dependent ROC analyses were performed. In here, a higher AUC values indicates the better staging system. Similarly, the seventh edition was identified as retaining its superior prognostic ability from the first to the tenth postoperative years (Table 5). In addition, as from the fifth postoperative year, the eighth edition was found to be superior compared to the fifth/sixth edition. However, clinically, these rankings demonstrated no significant influence due to the consistent overlapping of the 95% CI values calculated by their respective AIC, BIC, and AUC.

**Table 5:** AUC by the time‐dependent ROC analyses based on the predictive value of the multivariate analyses of the fifth/sixth, seventh, and eighth editions

Months	Fifth/Sixth edition		Seventh edition		Eighth edition	
	AUC	95% CI	AUC	95% CI	AUC	95% CI
12	0.765	0.730–0.800	0.767	0.728–0.802	0.763	0.730–0.794
24	0.803	0.776–0.826	0.803	0.779–0.827	0.796	0.770–0.817
36	0.793	0.769–0.813	0.797	0.767–0.821	0.790	0.764–0.813
48	0.773	0.743–0.795	0.780	0.756–0.806	0.772	0.744–0.796
60	0.765	0.736–0.793	0.774	0.751–0.801	0.768	0.740–0.790
120	0.782	0.754–0.805	0.790	0.762–0.822	0.786	0.756–0.808

AUC, area under curve; ROC, receiver operating characteristic; CI, confidence interval.

## Discussion

Most studies previously comparing the different GC staging editions were mainly based on simple analysis of HR values, AIC or BIC without further validating their results using other different statistical methods. In that, we felt the need for this extensive analysis by comparing their AIC and BIC values to confirm the calculated prognostic rankings since both methods have different statistical strengths 13.

Our results demonstrated that both statistical methods showed similar ranking, for which the seventh edition was identified as having the best predictive ability in both short‐ and long‐term despite the small numerical differences in allocating the ranks between the competing editions. In addition, the author hypothesized that since every classification constitutes of multiple subgroups, each of them might have their own predictive power. Therefore, a range of values (e.g., confidence intervals) would be more clinically reliable than an overall value (e.g., AIC, BIC) in the sense that the former would show the predictive range of each subgroups while the latter would simply depict an overall power for the whole group. Thereby, solely relying upon the raw values of AIC and BIC may not suffice for application in clinical practice 14. Consequently, their corresponding confidence intervals were calculated and considerable overlapping was found; which implied 15 that neither clinical nor statistical significance was reached to differentiate performance superiority among them and thus, signified that they possess similar prognostic ability.

Regarding the pTNM classification, the author suggests that the pT and pN should be compared as a combination because only as such they do correlate best to their corresponding overall survival (i.e., the overall survival of patients with pT2N0 will be different from those with pT2N3b). Otherwise, they may illustrate misleading, nonuniform prognoses with increase in disease severity, for example, in Table 1 for the seventh/eighth edition, pT classification showed a noncontinuous decrease in survival. Therefore, compared to previously published studies 16, 17, 18, we opted to assess the prognostic power between the stages of the different editions rather separately analyzing pT and pN. Subsequently, our results more illustratively demonstrated an improving homogeneity and distinctiveness between the successive stage groups of the different staging editions (Table 2).

Moreover, to achieve quality cancer care, choosing the optimal treatment for patients in different disease categories might be challenging, and these concerns have been gradually addressed by the AJCC. First, they discontinued the anatomical LN staging in the fifth edition. Second, they classified patients with distant metastasis separately as stage IV in the seventh edition. Then, they separated the pN3ab subgroup to pN3a and pN3b in the latest eighth edition main stage classifications. Progressively, these changes have resulted in providing a wider range of survival from stage IA to IIIC; as illustrated in Figures 2 and 3, the survival curve of stage IIIC for the eighth edition is noticeably lower than that of stage IIIC and stage IV in the seventh and fifth/sixth edition, respectively. Therefore, the eighth edition was identified as possessing the best discriminative ability for prognostic stratification of patients with gastric cancer and this will facilitate the identification of patients with higher‐stage disease for optimal therapies or enrollment in clinical trials, as the more advanced lesions have higher likelihood of nodal involvement, distant spread, recurrence and worse prognosis 19. Therefore, this recently revised edition, the eighth AJCC GC staging system, is a fundamental update and can be considered clinically more reliable than its previous versions.

This study is the most comprehensive one to evaluate the differences between the AJCC gastric cancer staging editions of the past two decades. However, it was limited by the fact that patients with gastric adenocarcinoma invading the EGJ could not be included for homogeneity of surgical treatment, as most of such cases were not operated by the same group of surgeons. Additionally, despite having a significant cohort of patients with R0 resections, due to greater subdivisions of classification in the eighth edition, the number of cases with pT2 disease was limited, however, since the new staging system mainly concerned patients with higher staged disease, this limitation strength was not significant to affect the statistical results of this study.

In conclusion, our comprehensive statistical assessment demonstrated that the eighth AJCC GC edition possess similar prognostic ability as the seventh, sixth, and fifth editions, which remained consistent in the long term. In addition, this new edition was also shown to provide the best discriminative ability and can thus serve as the new benchmark to stratify gastric cancer patients with higher stage disease.

## Conflict of Interest

All authors have signed the Form for Disclosure of Potential Conflicts of Interest, and no conflicts of interest were reported.
